# The Diagnostic Potential of *SHOX2* and *RASSF1A* DNA Methylation in Early Lung Adenocarcinoma

**DOI:** 10.3389/fonc.2022.849024

**Published:** 2022-06-28

**Authors:** Hong Gao, Jun Yang, Lu He, Wei Wang, Yanhong Liu, Yue Hu, Meiling Ge, Jie Ding, Qing Ye

**Affiliations:** ^1^Department of Pathology, Nanjing Drum Tower Hospital, The Affiliated Hospital of Nanjing University Medical School, Nanjing, China; ^2^Biobank of Nanjing Drum Tower Hospital, The Affiliated Hospital of Nanjing University Medical School, Nanjing, China; ^3^Department of Pathology, The First Affiliated Hospital of USTC, Division of Life Sciences and Medicine, University of Science and Technology of China, Hefei, China; ^4^Intelligent Pathology Institute, Division of Life Sciences and Medicine, University of Science and Technology of China, Hefei, China

**Keywords:** DNA methylation detection, *shox2*, *RASSF1A*, early lung adenocarcinoma, folate acid metabolism, DNA instability, tumor microenvironment

## Abstract

**Objective:**

Methylation of the promoters of *SHOX2* and *RASSF1A* are potentially informative biomarkers for the diagnosis of early lung adenocarcinoma (LUAD). Abnormal methylation of *SHOX2* and *RASSF1A* promoters may promote the occurrence and facilitate the progression of LUAD.

**Materials and Methods:**

We selected 54 patients with early LUAD and 31 patients with benign lung nodules as a NJDT cohort and evaluated their DNA methylation and mRNA sequencing levels. The DNA methylation sequencing, mRNA sequencing, and clinical data for patients with LUAD were obtained from The Cancer Genome Atlas, and served as a TCGA cohort. We evaluated the diagnostic potential of a *SHOX2* and *RASSF1A* combined promoter methylation assay for detection of early LUAD in the NJDT cohort. Then we explored the promoter methylation levels of *SHOX2* and *RASSF1A* and their gene expression between normal and tumor samples at different stages in both cohorts. Pathways enriched between tumor and normal samples of methylation-positive patients in the NJDT cohort were analyzed.

**Results:**

In the NJDT cohort, the sensitivity of the combined promoter methylation assay on tumor samples was 74.07%, the sensitivity on paired tumor and paracancerous samples was 77.78%, and the specificities in both contexts were 100%. The combined promoter methylation-positive patients had clinicopathologic features including older age, larger tumors, deeper invasion, and higher Ki-67 expression. In both cohorts, *SHOX2* expression increased and *RASSF1A* expression decreased in tumor samples. The promoter methylation level of *SHOX2* and *RASSF1A* was significantly higher in tumor samples at stage I-II than that in normal samples. The promoter methylation levels of these two genes were both negative associated with their expression in early tumor samples. In the NJDT cohort, methylation-positive patients of both individual *SHOX2* and *RASSF1A* assays exhibited upregulation of folate acid metabolism and nucleotide metabolism in tumor samples. The *SHOX2* methylation-positive and *RASSF1A* methylation-positive patients showed the downregulation of pathways related to cell proliferation and apoptosis and pathways involved in DNA repair, cell growth and cell adhesion, respectively.

**Conclusion:**

The combined promoter methylation assay for *SHOX2* and *RASSF1A* can be used for screening and diagnosis of early LUAD, with good sensitivity and specificity. The promoter methylation levels of *SHOX2* and *RASSF1A* were associated with their abnormal mRNA expression, and affected DNA instability, cell proliferation, apoptosis and tumor microenvironment in patients with LUAD.

## 1 Introduction

According to the World Health Organization (WHO), lung cancer is the leading cause of cancer death worldwide, with a morbidity rate of 11.4% and a mortality rate of 18.0% (1). In China, lung cancer has the highest incidence among malignant tumors (2). Lung adenocarcinoma (LUAD) is the most common histological subtype of non-small cell lung cancer (NSCLC), and accounts for ~40% of lung cancer cases. The surgical resection of the early-stage NSCLC offers a favourable prognosis, with 5-year survival rates of 70-90% (stage I), while most patients (approx. 75%) have advanced disease at the time of diagnosis (stage III/IV) and their survival remains poor (3). As sequencing techniques have developed, abnormal DNA methylation patterns have been found in various tumors, and are considered to be important causes of cancers (4). Methylation is often present in highly and moderately duplicated DNA sequences and plays a key role in chromosomal instability (5, 6). Promoter hypermethylation of tumor suppressor genes is usually associated with gene silencing (7). DNA methylation is involved in tumor formation in the early stages of carcinogenesis (8). In addition, DNA methylation is relatively stable over time and can be detected noninvasively in blood, urine, saliva and other body fluids. Therefore, more and more methylation biomarkers are being developed for early screening and diagnosis of tumors (9).

The detection of methylation patterns in Short Stature Homeobox 2 (*SHOX2*) and Ras-association domain family member 1A (*RASSF1A*) have been preliminarily used for the diagnosis of lung cancer. By comparing the methylation of *SHOX2* in lung cancer and normal tissues, ninety-six percent (53 out of 55) of matched pairs showed a higher methylation level in tumor tissues (10). The promoter region of *RASSF1A* is hypermethylated in 63% of NSCLC cell lines, but not in normal epithelial cells (11). In BALF, the sensitivity of the *SHOX2* and *RASSF1A* combined promoter methylation assay for NSCLC reached 71.5-83.2% and the specificity achieved 90.0-97.4% (12, 13). Moreover, the diagnostic value of the combined promoter methylation detection assay of *SHOX2* and *RASSF1A* for early LUAD has not been fully developed, and the mechanism by which the hypermethylation of *SHOX2* and *RASSF1A* contributes to LUAD occurrence and progression remains to be elucidated. Here we evaluated the significance of promoter methylation of *SHOX2* and *RASSF1A* in the diagnosis of early LUAD. Then we analyzed methylation data from different cohorts and we explored mechanisms of hypermethylated *SHOX2* and *RASSF1A*, leading to tumorigenesis and progression of LUAD.

## 2 Patients and Methods

### 2.1 The Recruitment Patients and Samples in the NJDT Cohort

A total of 54 patients with early LUAD and 31 patients with benign lung nodules who underwent surgeries in Nanjing Drum Tower Hospital from January 2017 to January 2018 were recruited (NJDT cohort). All patients had signed informed consent for dominating their samples. Preoperative computed tomography (CT) scan results of all patients indicated pulmonary nodules, and postoperative pathological diagnosis indicated LUAD or benign lung tumors (pulmonary atypical adenomatous hyperplasia, pulmonary fibrosis nodules, and pulmonary inflammatory pseudotumors). The matched samples of tumor, paracancerous (distance from tumor less than 1 cm) and normal lung tissue (distance from tumor more than 5 cm) were collected from each patient with LUAD. The matched samples of nodule, perinodular (distance from nodule less than 1 cm) and normal lung tissue (distance from tumor more than 5 cm) were collected from each patient with benign lung nodules. No patients received ablative therapy, chemotherapy, or radiation therapy before surgery. The sample collection and research were approved by the Ethics Committee of Nanjing Drum Tower Hospital.

### 2.2 Sample Examination in the NJDT Cohort

#### 2.2.1 Pathological Evaluation

Formalin-fixed paraffin-embedded (FFPE) samples of patients with LUAD and patients with benign lung nodules were collected. The paraffin-embedded samples were cut into pathological sections, stained by hematoxylin and eosin (HE), and examined by two pathologists. The samples were graded and classified according to TNM Stage Groupings in the Eighth Edition proposed by International Association for the Study of Lung Cancer (IASLC) and the 2015 World Health Organization (WHO) Classification of Tumors of the Lung, Pleura, Thymus and Heart. In view of the high heterogeneity of lung adenocarcinoma, the major histological classification of each sample was determined by the dominant component. The clinicopathological data of the patients are shown in **Table 1**.

**Table 1:** The clinicopathologic characteristics of patients with LUAD and pulmonary benign nodules in the NJDT cohort.

	LUAD	Benign nodules	*P*
**Age (n=54,31)**			
	61.02 ± 10.1 6(27-84)	55.47 ± 7.91 (23-67)	0.1174
**Sex (n=54,31)**			
Male	31	16	0.554
Female	22	15	
**MTD (n=54,31)**			
	1.73 ± 0.137	2.147 ± 0.225	0.2398
**Pathological types of benign nodules (n=31)**			
AAH	NA	12 (38.71)	NA
Fibrosis Nodules	NA	13 (41.94)	
Inflammatory Pseudotumors	NA	4 (12.90)	
Others	NA	2 (6.45)	
**Differentiation of LUAD (n=47)**			
Low, n (%)	7 (14.90)	NA	NA
Medium, n (%)	20 (42.55)	NA	
High, n (%)	20 (42.55)	NA	
**TNM stages of LUAD (n=54)**			
0(Tis), n (%)	3 (5.56)	NA	NA
IA, n (%)	36 (66.67)	NA	
IB, n (%)	8 (14.81)	NA	
II, n (%)	7 (12.96)	NA	
**Pathological types of LUAD (n=54)**			
AIS, n (%)	3 (5.56)	NA	NA
MIA, n (%)	10 (18.52)	NA	
IPA, n (%)	41 (75.92)	NA	

MTD, maximum tumor diameter; AAH, atypical adenomatous hyperplasia; AIS, adenocarcinoma in situ; MIA, minimally invasive adenocarcinoma; IPA, Invasive pulmonary adenocarcinoma. NA, not available.

#### 2.2.2 DNA Extraction and Bisulfite Treatment

Genomic DNA (gDNA) was extracted from FFPE samples using E.Z.N.A FFPE DNA Kit (Omega, Shanghai, China) according to the manufacturer’s instructions. The gDNA was treated with the EZ DNA Methylation Kit (Zymo Research, Beijing, China), according to the manufacturer’s instructions. This technique involves treating methylated DNA with bisulfite, which converts unmethylated cytosines into uracil, while, methylated cytosines remain unchanged during the treatment.

#### 2.2.3 Methylation Detection and Analysis

The commercial *SHOX2* and *RASSF1A* Methylation Detection Kit (Tellgen, Shanghai, China) for lung cancer was used to detect the methylation levels of CpG islands (CGIs) in the *SHOX2* and *RASSF1A* promoter regions (13). Methylated *SHOX2* and *RASSF1A* DNA plasmids were used as controls. A Roche LightCycler 480 II Real-time PCR System was used for quantitative real-time PCR (qPCR). The result interpretation of qPCR was carried out according to manufacturer’s instructions. An amplification curve of the FAM fluorescence signal with a smooth “S” shape and a threshold cycle (C_T_) < 35 indicated a positive result for *RASSF1A* methylation [*RASSF1A*_met (+)]; C_T_ ≥ 35 indicated a negative result for *RASSF1A* methylation [*RASSF1A*_met (-)]. An amplification curve of the VIC fluorescence signal with a smooth “S” shape and a C_T_ < 32 indicated a positive result for *SHOX2* methylation [*SHOX2*_met (+)]; a C_T_ ≥ 32 indicated a negative result for *SHOX2* methylation [*SHOX2*_met (-)]. Either a positive *RASSF1A* or positive *SHOX2* methylation result indicated a positive combined methylation result [combination_met (+)]; when both the *RASSF1A* and *SHOX2* methylation results were negative, the result of the combined methylation test was negative [combination_met (-)].

#### 2.2.4 Immunohistochemical Detection Interpretation

The tumor, paracancerous and normal FFPE samples in the NJDT cohort were cut into pathological sections and evaluated by immunohistochemistry (IHC). The IHC analyses were performed using rabbit anti-human polyclonal antibodies against Ki-67 (Thermo Fisher Scientific, catalog # MA5-14520, RRID AB_10979488), TTF-1 (Thermo Fisher Scientific, catalog # PA5-78209, RRID AB_2736758), Napsin A (Thermo Fisher Scientific, catalog # PA5-60970, RRID AB_2644471) as primary antibodies, and the goat anti-rabbit polyclonal antibody as the secondary antibody. Known positive sections were used as positive controls, and sections treated by PBS instead of primary antibody were used as negative controls. The IHC results were evaluated according to the staining intensity and percentage of positive tumor cells, (1) Napsin A IHC results were interpreted as follows (14): ① Based on the percentage of positive cells, 0 point for no positive cells, 1 point for the percentage of positive cells < 25%, 2 points for percentage of positive cells between 25% and 49%, and 3 points for percentage of positive cells ≥ 50%; ② Based on the staining intensity, 0 point for no staining, 1 point for light yellow staining, 2 points for moderate yellow staining, 3 points for brown staining. The product of ① and ② was regarded as the immunohistochemical score (IHCS). An IHCS < 3 was interpreted as Napsin A negative. An IHCS≥3 was interpreted as Napsin A positive. (2) Ki-67 IHC results were based on percentage of positive cells (15): a percentage of positive cells <10% indicated Ki-67 negative; a percentage of positive cells ≥10% indicated Ki-67 positive. (3) TTF-1 IHC results were based on staining intensity (16): staining with no color or light yellow indicated TTF-1 negative; staining with moderate yellow or brown indicated TTF-1 positive.

#### 2.2.5 mRNA Library Construction and Sequencing

The FFPE tumor and paired normal samples of 45 patients were selected from 54 patients with LUAD for mRNA sequencing. The percentage of tumor cells in these samples should be more than 80%. Among the patients, 25 out of 45 were tested positive for *SHOX2* promoter methylation and 18 out of 45 were tested positive for *RASSF1A* promoter methylation. Total RNA from samples was extracted using miRNeasy FFPE kit (QIAGEN). Ribosomal RNA was depleted using KAPA Stranded RNA-seq Kit with RiboErase (HMR) (KAPA Biosystems). Library preparations were performed with KAPA Stranded RNA-seq Library Preparation Kit (Roche). Library concentration was determined by KAPA Library Quantification Kit (KAPA Biosystems), and library quality was accessed by Agilent High Sensitivity DNA kit on Bioanalyzer 2100 (Agilent Technologies), which was then sequenced on Illumina HiSeq NGS platforms (Illumina). The amount of sequencing data for each sample was 30M.

#### 2.2.6 mRNA Sequencing Data Analysis

The high-quality reads generated were aligned to the human reference genome (UCSC hg19) with hisat2 software. Then, guided by the Ensembl gene-annotation file, cuffdiff software (part of cufflinks) was used to reveal the expression profile of the mRNAs in terms of Fragments Per Kilobase of transcript per Million mapped reads (FPKM) values. The FPKM values were used to for the analysis of gene expression and enriched pathways. The Gene Set Enrichment Analysis (GSEA) algorithm was used to analyze differentially enriched pathways between tumor and matched normal tissues in *SHOX2*_met (+) and *RASSF1A*_met (+) groups, respectively (17, 18). The enrichment pathways were sorted by nom *P*-value and normalized enrichment score (NES), and a false discovery rate (FDR) value was determined. When |NES| > 1, nom P-value < 0.05, and FDR < 25%, the enriched pathways were significantly different between the tumor and normal samples.

### 2.3 Data Acquisition and Analysis of the TCGA Cohort

DNA methylation sequencing data from 465 LUAD samples and 31 normal samples were downloaded from the TCGA (https://portal.gdc.cancer.gov/). The beta values (β) were used to indicate the methylation level of methylated cytosine-guanine (CpG) dinucleotides. The mRNA sequencing data (HTSeq-FPKM) of 526 LUAD samples and 59 normal samples were also downloaded. The only CGI in the promoter region of *SHOX2* contains six CpG sites, including cg01557547, cg04532033, cg06156376, cg16703882, cg18899952 and cg25694447. The only CGI in the promoter region of *RASSF1A* contains eleven CpG sites, including cg00777121, cg04743654, cg06172942, cg08047457, cg12966367, cg13872831, cg21554552, cg24859722, cg25486143 and cg25747192. The average normalized levels of the CpG sites in the promoter region were calculated as the CGI levels of *SHOX2* and *RASSF1A*, respectively.

### 2.4 Workflow

**Figure 1:**
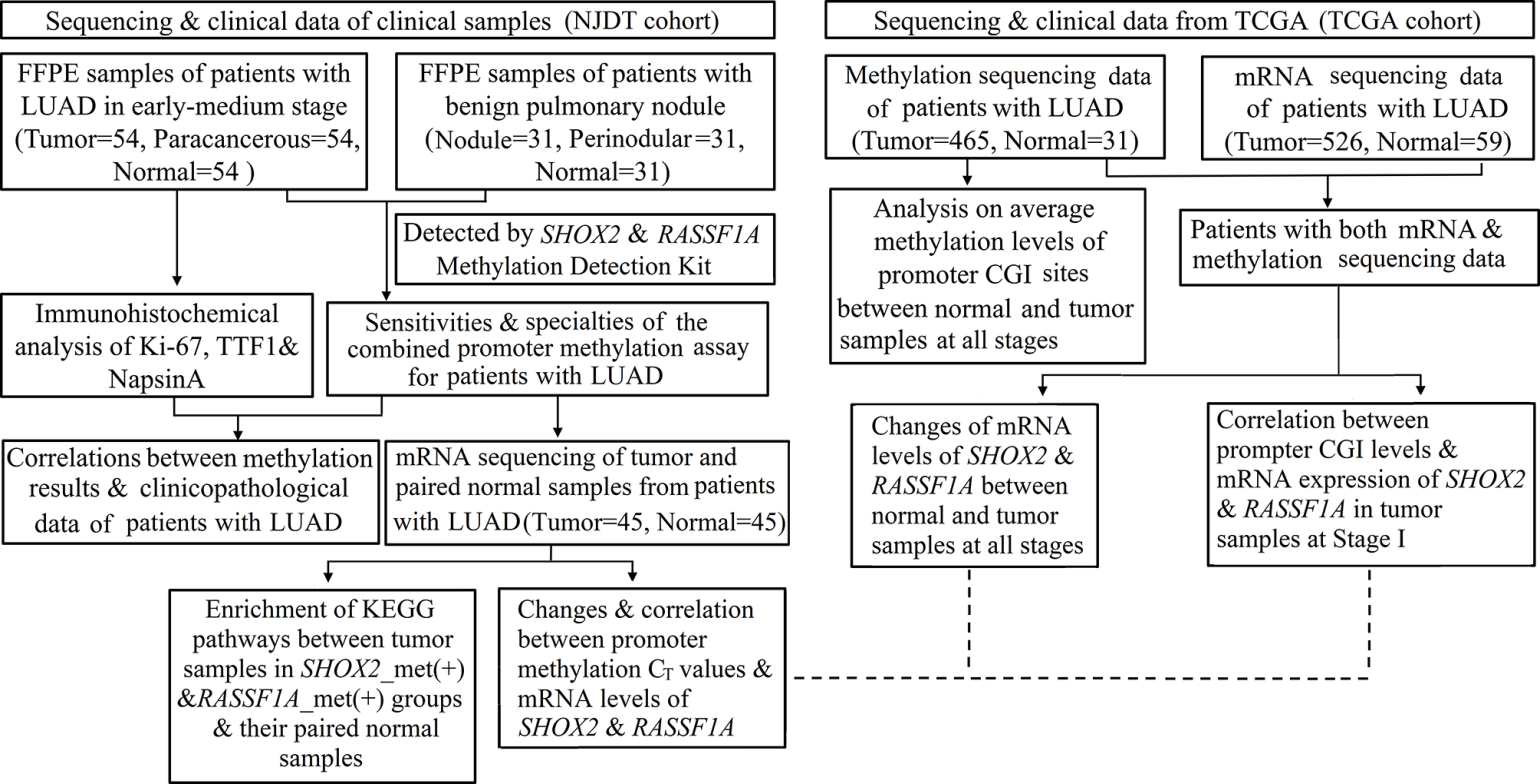
Flowchart of the study protocol. LUAD, early lung adenocarcinoma; FFPE, Formalin Fixed and Paraffin Embedded tissues; ROC, Receiver operating characteristics; CGIs, CpG islands; *SHOX2*_met (+), positive results of *SHOX2* promoter methylation assay; *RASSF1A*_met (+), positive results of *RASSF1A* promoter methylation assay.

The workflow of this study is demonstrated in **Figure 1**,

## 3 Statistical Analysis

The statistical analyses were conducted with R software (version 4.0.2), GraphPad Prism software (version 6.0) and SPSS software (version 19.0). Receiver Operating Characteristics (ROC) curves were constructed to explore the diagnostic ability of the combined promoter methylation assay of *SHOX2* and *RASSF1A* for early LUAD patients and calculate the specificities (SPs), and sensitivities (SEs). The DeLong test was used to evaluate the area under curves (AUC). The independent t-test was used for the comparison of continuous clinical variables and the Chi-square test or Fisher’s exact test was used to compare discontinuous clinical variables between combination_met (+) and combination_met (-) groups. Wilcoxon test was used to compare the methylation level between normal and tumor samples at different stages. Spearman correlation analysis was used to compute the correlation between methylation levels and gene expression in both cohorts. The multiple hypothesis test with the Benjamini-Hochberg method was used to control false discovery rate (FDR). All statistical tests were two-sided, and *P* values less than 0.05 were considered statistically significant (*P < 0.05, **P < 0.01, ***P < 0.001).

## 4 Results

### 4.1 The Diagnostic Value of the *SHOX2* and *RASSF1A* Combined Promoter Methylation Assay for Patients With LUAD in the NJDT Cohort

**Figure 2:**
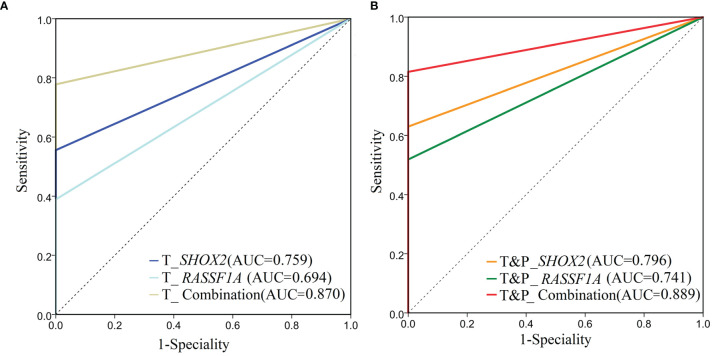
ROC analysis of *SHOX2*, *RASSF1A* and the combined promoter methylation assays on patients in the NJDT cohort. **(A)** ROC analysis of *SHOX2*, *RASSF1A*, and the combined promoter methylation assays of tumor samples in the NJDT cohort. T_*RASSF1A*: *RASSF1A* methylation assay on tumor samples; T_*SHOX2*: *SHOX2* methylation assay on tumor samples; T_Combination: The combined promoter methylation assay of *SHOX2* and *RASSF1A* on tumor samples; **(B)** ROC analysis of *SHOX2*, *RASSF1A*, and the combination methylation assays on tumor and matched paracancerous (T&P) samples in the NJDT cohort. T&P _*RASSF1A*: *RASSF1A* methylation assay on tumor and matched paracancerous samples; if either kind of samples were positive, the results were considered positive; T&P_*SHOX2*: *SHOX2* methylation assay on tumor and matched paracancerous samples; if either kind of samples were positive, the results were considered positive; T&P_Combination: The combined promoter methylation assay of *SHOX2* and *RASSF1A* on tumor and matched paracancerous samples; if either kind of samples were positive, the results were considered positive.

The *SHOX2* and *RASSF1A* combined promoter methylation assay was performed on samples from 54 patients with early LUAD and 31 patients with benign lung nodules. The positive cases of the individual *SHOX2*, individual *RASSF1A* and combined promoter methylation assays were shown in the supplementary table (**Supplementary Table 1**). For the tumor samples from patients with LUAD, the sensitivity of the *SHOX2* promoter methylation assay was slightly higher than that of *RASSF1A*, while the AUC of the *SHOX2* and *RASSF1A* combined promoter methylation assay was significantly higher than those of individual *SHOX2* and *RASSF1A* assays, respectively (DeLong test*, P* < 0.05) (**Table 2**; **Figure 2A**). The individual *SHOX2* and *RASSF1A* assays were also sensitive to paracancerous samples of LUAD. 4/54 patients with LUAD were positive in paracancerous samples but negative in tumor ones detected by the individual *SHOX2* assay. 5/54 patients with LUAD were positive in paracancerous samples but negative in tumor ones detected by the individual *RASSF1A* assay (**Supplementary Figure 1**). The tumor and matched paracancerous (T&P) samples were evaluated by the combined promoter methylation assay. The result was considered positive if either the tumor sample or the paracancerous sample was positive. The combined assay had higher sensitivity on T&P samples than on tumor samples from LUAD patients, and its AUC was significantly higher than those of individual *SHOX2* and *RASSF1A* assays, respectively (DeLong test*, P* < 0.05) (**Table 2**; **Figure 2B**). In addition, the specificities of the individual and combined assays were all 100% for LUAD patients in the NJDT cohort.

**Table 2:** The sensitivities and specialties of *SHOX2*, *RASSF1A* and the combined promoter methylation assays on patients in the NJDT cohort.

Group	AUC (95% CI)	Sensitivity% (SE)	Specificity% (SP)
T_*SHOX2*	0.759 (0.654~0.845)^$^	51.85 (37.8~65.7)	100.00 (88.8~100.0)
T_ *RASSF1A*	0.694 (0.585~0.790)^#^	38.89 (25.9~53.1)	100.00 (88.8~100.0)
T_ Combination	0.870 (0.780~0.933)^$,#^	74.07 (60.3~85.0)	100.00 (88.8~100.0)
T&P _ *SHOX2*	0.796 (0.695~0.876)^£^	59.26 (45.0~72.4)	100.00 (88.8~100.0)
T&P _ *RASSF1A*	0.741 (0.634~0.830)^§^	48.15 (34.3~62.2)	100.00 (88.8~100.0)
T&P_ Combination	0.889 (0.802~0.947)^£,§^	77.78 (64.6~88.7)	100.00 (88.8~100.0)

T_RASSF1A: RASSF1A promoter methylation assay on tumor samples; T_ SHOX2: SHOX2 promoter methylation assay on tumor samples; T_Combination: The combined promoter methylation assay of SHOX2 and RASSF1A on tumor samples; T&P _ RASSF1A: RASSF1A promoter methylation assay on the tumor and matched paracancerous samples, either kind of samples was positive, the result was positive; T&P_ SHOX2: SHOX2 promoter methylation assay on the tumor and matched paracancerous samples, either kind of samples was positive, the result was positive; T&P_Combination: The combined promoter methylation assay of SHOX2 and RASSF1A on the tumor and matched paracancerous samples, either kind of samples was positive, the result was positive. The superscripts symbols "$, #, £ and §" represent that the AUCs of the two groups with the same symbol were compared and showed to be significantly different, respectively (P < 0.05).

### 4.2 Comparison of Clinicopathological Characteristics of Patients Identified as Methylation Positive or Negative by the Combined *SHOX2* and *RASSF1A* Promoter Methylation Assay

**Figure 3:**
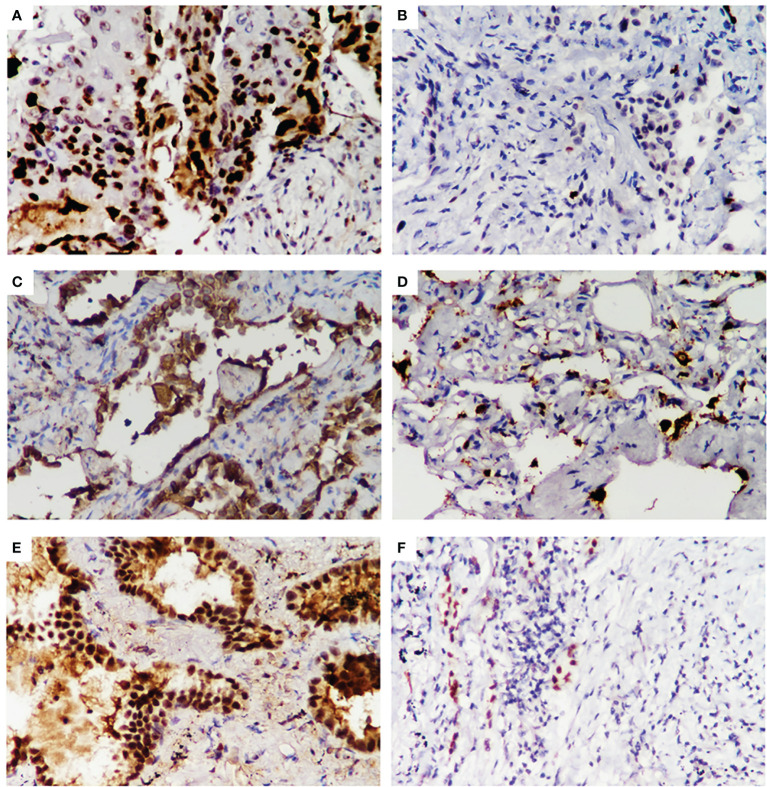
Immunohistochemical staining for Ki-67, Napsin A, and TTF1 expression in early LUAD samples from the NJDT cohort. Positive results (×400) of Ki-67**(A)**, Napsin A (**C**), TTF1 (**E**) expression FFPE samples of early LUAD by IHC analysis. Negative results (×400) of Ki-67 (**B**), Napsin A **(D)**, and TTF1 **(F)** expression FFPE samples of early LUAD by IHC analysis.

We compared the clinicopathological features between LUAD patients identified as combination_met (+) or combination_met (-) by the combined promoter methylation assay from the NJDT cohort (**Supplementary Table 2**; **Supplementary Figure 2**). The patients in the combination_met (+) group were characterized by older age (Independent t-test, *P* < 0.05), larger tumor size (Independent t-test, *P* < 0.05), invasive adenocarcinoma subtype (Fisher’s exact test, *P* < 0.05), and advanced TNM stages (Fisher’s exact test, *P* < 0.05) (**Table 3**). None of the 3 patients with Stage 0 LUAD were classified as combination_met (+). In the Stage IA group, 72% of the patients were classified as combination_met (+), while the percentage classified as combination_met (+) in the Stage IB and II groups were 100% and 86%, respectively. In patients with early LUAD from Stage 0 to Stage II, the positive rates of the *SHOX2* and *RASSF1A* methylation assay increased significantly along with progression of disease stage. As the pathological subtype progressed from AIS to MIA to IPA, the percentage of combination_met (+) cases also significantly rose (Fisher’s exact test, *P* < 0.05). In addition, we evaluated Ki67, TTF-1, and Napsin A expression in LUAD samples by IHC analysis (**Figure 3**). The combination_met (+) group had more patients with positive Ki67 expression by IHC than those in the combination_met (-) group (Chi-square test, *P* < 0.05), but TTF-1 and Napsin A did not show the phenomenon (Chi-square test, *P* > 0.05) (**Table 3**).

**Table 3:** Clinicopathologic characteristics between patients of combination_met (+) and combination_met (-) groups in the NJDT cohort.

	combination*_*met (+)		combination*_*met (-)	*P*
**Sex (n=54), n (%)**				
Male	23(74.19)		8(25.81)	0.473
Female	17(73.91)		6(26.09)	
**Age (n=54)**				
	64.15 ± 8.71		55.01 ± 12.84	0.014*
**MTD (n=54)**				
	2.29 ± 1.05		1.68 ± 1.10	0.024*
**Histological subtypes (n=54), n (%)**				
LPA	20(71.43)		8(28.57)	0.335
APA	13(72.22)		5(27.78)	
PPA	2(66.67)		1(33.33)	
Others	5(100.00)		0(0.00)	
**Pathological type (n=54), n (%)**				
AIS	0(0.00)		3(100.00)	<0.001***
MIA	5(50.00)		5(50.00)	
IPA	35(85.37)		6(14.63)	
**Differentiation (n=47), n (%)**				
Low	6(85.71)		1(14.29)	0.208
Medium	17(85)		3(15)	
High	12(60)		8(40)	
**TNM stages (n=54), n (%)**				
Stage 0	0(0.00)		3(100.00)	0.009**
Stage I	34(77.27)		10(22.73)	
Stage II	6(85.71)		1(14.29)	
**T classification (n=54), n (%)**				
Tis	0(0)		3(100)	0.007**
T1	29(74.36)		10(25.64)	
T2	11(91.67)		1(8.33)	
**Lymphatic metastasis (n=54), n (%)**				
Positive	2(100)		0(0)	0.436
Negative	38(73.08)		14(26.92)	
**Ki67 (n=47), n (%)**				
Positive	19(95)		1(5)	0.016*
Negative	16(59.26)		11(40.74)	
**TTF1 (n=47), n (%)**				
Positive	22(84.62)		4(15.38)	0.142
Negative	13(61.9)		8(38.1)	
**Napsin A (n=47), n (%)**				
Positive	18(72)		7(28)	0.585
Negative	17(77.27)		5(22.73)	

MTD, maximum tumor diameter; AIS, adenocarcinoma in situ; MIA, minimally invasive adenocarcinoma; IPA, Invasive pulmonary adenocarcinoma; LPA, lepidic predominant adenocarcinoma; APA, acinar predominant adenocarcinoma; PPA, papillary predominant adenocarcinoma. ∗P < 0.05, ∗∗P < 0.01, ∗∗∗P < 0.001.

### 4.3 Changes and Correlation Between Promoter Methylation and mRNA levels of *SHOX2* and *RASSF1A* in Both Cohorts

**Figure 4:**
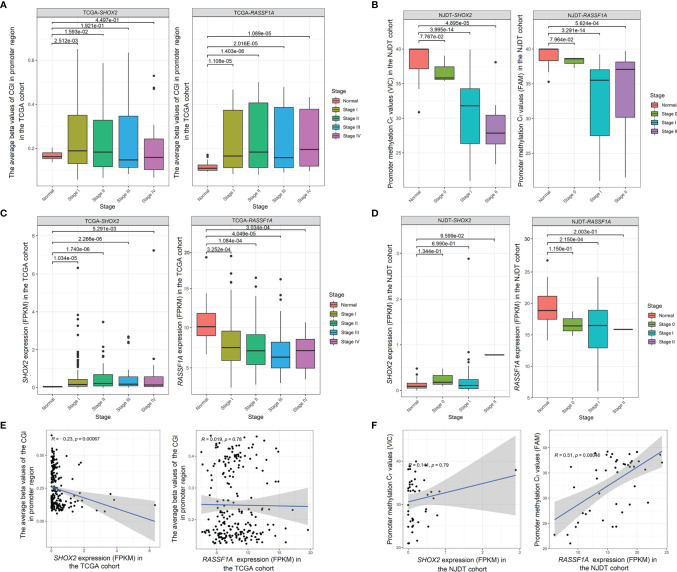
Changes and correlation between promoter methylation and mRNA levels of *SHOX2* and *RASSF1A* in both cohorts **(A)** Comparison of the promoter CGI levels of *SHOX2* (left) and *RASSF1A* (right) between normal samples and tumor samples at Stage I-IV from the TCGA cohort; **(B)** Comparison of the promoter methylation C_T_ values of *SHOX2* (left) and *RASSF1A* (right) betwwen normal samples and tumor samples at Stage 0-II from the NJDT cohort. The C_T_ values which were not detected within 40 cycles (>40) were calculated as 40 in the normal samples; **(C)** Comparison of the expression (FPKM values) of *SHOX2* (left) and *RASSF1A* (right) between normal samples and tumor samples at Stage I-IV from the TCGA cohort; **(D)** Comparison of the expression (FPKM values) of *SHOX2* (left) and *RASSF1A* (right) between normal samples and tumor samples at Stage 0-II from the NJDT cohort; **(E)** Correlation between promoter CGI levels and mRNA levels of *SHOX2* (left) and *RASSF1A* (right) in tumor samples at Stage I from the TCGA cohort; **(F)** Correlation between the promoter methylation C_T_ values and mRNA levels of *SHOX2* (left) and *RASSF1A* (right) in tumor samples from the NJDT cohort.

The DNA methylation and mRNA sequencing data from the TCGA cohort were used to explore changes and correlation between promoter methylation and mRNA levels of *SHOX2* and *RASSF1A* (**Supplementary Tables 3, 4**). The promoter CGI levels of *SHOX2* in LUAD samples at Stage I and II were significantly higher than those of normal samples (Wilcoxon test, *P* < 0.05), but samples at Stage III and IV showed no significance (Wilcoxon test, *P* > 0.05) (**Figure 4A**). While, the promoter CGI levels of *RASSF1A* maintained high at all stages of the disease (Wilcoxon test, *P* < 0.001) (**Figure 4A**). The expression of *SHOX2* was significantly higher in tumor samples at Stage I-II than that in normal samples, but there was a negative correlation between *SHOX2* expression and its promoter methylation level in tumor samples at Stage I (Spearman correlation, *P* < 0.05) (**Figure 4C**). The expression level of *RASSF1A* was significantly lower in tumor samples at all stages than that in normal samples, and the promoter methylation level of *RASSF1A* seemed negatively correlated with its expression in tumor samples at Stage I, but there was no significant difference (Spearman correlation, *P* > 0.05) (**Figure 4E**).

In the NJDT cohort, the promoter methylation C_T_ values of both *SHOX2* and *RASSF1A* in tumor samples at Stage I and II were significantly lower than those in normal samples (Wilcoxon test, *P* < 0.05) (**Figure 4B**). Compared with normal samples, the *SHOX2* expression showed a slight increase in tumor samples at Stage I (Wilcoxon test, *P* > 0.05) and a slight positive correction with the C_T_ values (Spearman correlation, *P* > 0.05) (**Figures 4D, F**). The expression of *RASSF1A* in tumor samples at Stage I was significantly lower than that in normal samples and showed a significantly positive correction with C_T_ values (Wilcoxon test, *P* < 0.05) (**Figure 4D, F**). Therefore, the promoter methylation of both *SHOX2* and *RASSF1A* in early tumor samples were negative associated with their expression, respectively.

### 4.4 KEGG Pathways Enrichment Analysis Between Normal and Tumor Samples in Methylation Positive Groups From the NJDT Cohort

**Figure 5:**
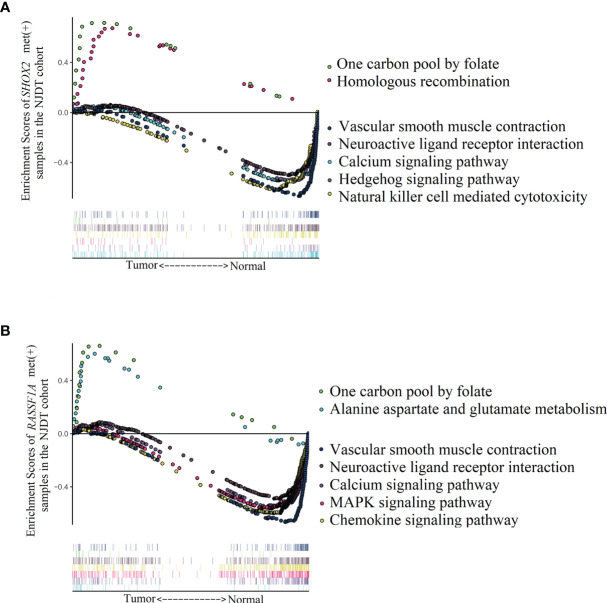
Enrichment of KEGG pathways analyzed by GSEA between LUAD and matched normal samples of *SHOX2*_met (+) and *RASSF1A*_met (+) groups in the NJDT cohorts **(A)** The significant upregulated pathways and top 5 downregulated pathways of *SHOX2*_met (+) LUAD samples; **(B)** The significant upregulated pathways and top 5 downregulated pathways of *RASSF1A*_met (+) LUAD samples.

In order to explore biological pathways that might be influenced by hypermethylation of *SHOX2* and *RASSF1A* in promoter regions, we performed KEGG pathway enrichment analysis by GSEA on the mRNA sequencing data from NJDT cohort (**Supplementary Table 5**). Compared with normal samples, *SHOX2*_met (+) tumor samples exhibited upregulation of two specific pathways related to folate metabolism (one carbon pool by folate) and DNA metabolism (homologous recombination) (**Figure 5A**). While, enriched pathways including vasoconstriction (vascular smooth muscle contraction, calcium signaling pathway), cell apoptosis and differentiation (TGF beta signaling pathway), signal transduction (neuroactive ligand receptor interaction), and water and salt metabolism (aldosterone regulated sodium reabsorption) were specifically downregulated in *SHOX2*_met (+) tumor samples. Meanwhile, *RASSF1A*_met (+) samples exhibited upregulation of two specific pathways which were related to folate metabolism (one carbon pool by folate) and cytosine synthesis (alanine aspartate and glutamate metabolism) (**Figure 5B**). While, enriched pathways of vasoconstriction (vascular smooth muscle contraction, calcium signaling pathway, regulation of actin cytoskeleton), gene transcription (WNT Signaling Pathway), cell differentiation and apoptosis (MAPK signaling pathway), signal transduction (neuroactive ligand receptor interaction), cell adhesion (cell adhesion molecules CAMs, tight junction, gap junction) and lipid metabolism (PPAR signaling pathway, adipocytokine signaling pathway) were significantly downregulated in *RASSF1A_*met (+) tumor samples. In the NJDT cohort, methylation-positive tumor samples of both individual *SHOX2* and *RASSF1A* assays showed common upregulation of folate metabolism and nucleotide metabolism and common downregulation of vasoconstriction, cell apoptosis and differentiation, and nutrition metabolism involved in tumor microenvironment.

## 5 Discussion

NSCLC makes up about 85% of newly diagnosed lung cancer cases, and LUAD is the most common type of new NSCLC, accounting for about 40%. Traditional screening methods for NSCLC include sputum cytology, chest radiography, and computed tomography (CT) (19). However, among the small pulmonary nodules detected by imaging, up to 96% are benign nodules. Developing effective genetic biomarkers to distinguish malignant from benign nodules will be very beneficial for accurate diagnosis and improved treatment (20). With the advancement of epigenetic research, the mechanisms by which epigenetic modifications, including DNA methylation, are involved in cancer pathogenesis and becoming better understood (21). *SHOX2* and *RASSF1A* methylation tests have diagnostic specificity and sensitivity in peripheral blood, alveolar lavage fluid, and tissue biopsy from lung cancer patients (12, 13), but their potential for screening and diagnosis of patients with early LUAD remains unclear.

In the present study, we examined matched tumor, paracancerous tissue, and normal samples from 54 patients with LUAD. We found that *SHOX2* or *RASSF1A* promoter methylation tests are sensitive and specific for early LUAD, but the diagnostic efficacy of individual gene methylation assays was not high. For tumor samples, nearly twenty-three percent (9 out of 40) of combination_met (+) patients were positive in both individual assays. While, the rest (31 out of 40) combination_met (+) patients were only positive in one individual assay (**Supplementary Figure 1**). Therefore, the detection of both two genes can compensate the sensitive range of each other to some extent. Since there were only 3 patients with Stage 0 in the NJDT cohort, and their combined promoter methylation assays were all negative, the diagnostic potential of the combined promoter methylation assay for patients with Stage 0 remains unclear. Nevertheless, the sensitivity of the combined promoter assay was improved when it was applied on paired tumor and paracancerous samples instead of on tumor samples alone. This phenomenon was only seen in LUAD patients, but not in patients with benign lung nodules. We suggest that hypermethylation often precedes tumor formation and may be present in both tumor area and the vicinity. However, in the early stage of lung adenocarcinoma, the tumor is still in the initial stage with small size, and the hypermethylation of some focal cells has not been completely formed. Due to the limitation of location and volume of sampling, unmethylated tumor cells were collected and resulted in false positive error. While, the positive rates of the vicinity made up for this loss. It also indicates that the combined assay can be used to improve the detectable rates of early LUAD for those BALF and sputum samples, in which only paracancerous cells were obtained.

Subsequently, we analyzed the clinicopathological features of patients with early LUAD in different groups. In the NJDT cohort, as the age and clinicopathologic stage increased, the percentage of methylation-positive patients increased. As disease stage increased from Stage 0-II, or as LUAD progressed from AIS to MIA to IPA, promoter methylation levels of *SHOX2* or *RASSF1A* increased gradually. We also found that the expression of Ki-67 positively correlates with the combined promoter methylation level of *SHOX2* and *RASSF1A*. This is clinically relevant, as the 3-year survival rate of patients with high expression of Ki-67 is lower than that of those without Ki-67 expression. In primary lung cancer, high Ki-67 expression is associated with increased proliferation cancer cells (22), poor disease-free survival rates, and is significantly correlated with brain metastasis (23). This suggests that patients who tested positive by the combined methylation assay, may have rapid tumor progression and need aggressive treatments, despite perhaps having early-stage LUAD. Additionally, patients who were negative by the combined methylation assay may have the disease with relatively slow tumor cell proliferation.

*SHOX2* is considered to be an oncogene in many published reports (24–27). We found higher levels of promoter methylation and gene expression in tumor samples, and the levels were associated negatively. This may indicate that promoter hypermethylation of *SHOX2* regulates its expression to a certain extent, but it is not the only regulatory mode, and there may be other ways leading to the upregulation of *SHOX2* in tumor samples. Furthermore, analysis on the TCGA cohort demonstrated that the methylation level of *SHOX2* has not significantly risen at Stage III-IV. It may suggest that *SHOX2* promoter hypermethylation is a biomarker for early LUAD but not for advanced LUAD. On the other hand, *RASSF1A* is considered to be a tumor suppressor gene (26, 28–30). In both cohorts, the promoter methylation level of *RASSF1A* were higher in tumor samples at all stages than those in normal samples, but its expression was lower. It seems that promoter hypermethylation and expression of *RASSF1A* can be used as biomarkers for early and advanced LUAD.

Although, the functions of *SHOX2* and *RASSF1A* in some cancer contexts have been reported, the role of *SHOX2* and *RASSF1A* in the occurrence and development of LUAD remain to be explored. In the NJDT cohort, compared with the matched normal samples, both the *SHOX2*_met (+) and *RASSF1A*_met (+) tumor samples had upregulation of pathways, which may be related to tumor DNA hypermethylation and instability. These positive samples were in a hypermethylated state, and the hypermethylation of these two genes was the embodiment of the hypermethylated of the whole genome. However, the hypermethylation of these two genes directly or indirectly affected the upstream and downstream carcinogenic pathways including apoptosis, DNA repair, and cell metabolism. In the meanwhile, *SHOX2*_met (+) tumor samples showed the downregulation of TGF beta signaling pathway, which is related to inhibiting tumor growth by triggering the cell stagnation and apoptosis, in the early stage of tumor formation (31). It is reported that *SHOX2* can restrain the expression of bone morphogenetic protein 4 (*Bmp4*) (32), and *Bmp4* indirectly inhibits the expression of RUNX family transcription factor 2 (*RUNX2*) (33). Therefore, the increase of *SHOX2* in tumor samples indirectly leads to the upregulation of *RUNX2*. *RUNX2* plays an important role in regulating cell and vascular growth and differentiation mediated by transforming growth factor-β (TGF-β) and vascular endothelial growth factor (VEGF) (34). In addition, the overexpression of *SHOX2* can enhance its functions of downregulating p53 activity, activating NF-κB to promote tumorigenesis and drug resistance and inhibiting apoptosis in lung cancer cells (35). In summary, *SHOX2* regulates the proliferation, apoptosis and metastasis of LUAD cells, and may facilitate pro-tumor biological processes. *RASSF1A*_met (+) samples showed downregulation of several important pathways, which were involved in DNA repair, gene transcription, cell adhesion, cell differentiation and apoptosis. The RASSF1A protein has an ataxia telangiectasia mutated (ATM) phosphorylation site, which helps to regulate phosphorylation of DNA damage checkpoints and participates in the regulation of genomic stability (36, 37). The loss of RASSF1A enhances TLR-driven NF-κB activation and induces inflammatory DNA damage (38). *RASSF1A* deletion reduces the expression of β-catenin and E-cadherin, leading to tumor cell migration and invasion (39). In addition, *RASSF1A* is also linked with MAPK signaling pathway. Currently, there are conflicting reports on the interaction between *RASSF1A* and MAPK. It has been suggested that *RASSF1A* competitively binds to MST2 in the RAF-1-MST2-inhibiting complex, thereby enhancing the activity of RAF-1 and the Ras-MAPK pathway (38, 40). It has also been reported that high expression of RASSF1A can inhibit the activation of extracellular regulated protein kinases 1/2 (ERK1/2) and reduce the activity of the RAS-MAPK pathway (41). While, our results suggested that decreased expression of *RASSF1A* in LUAD samples were related to downregulation of the Ras-MAPK pathway. However, there is no doubt that *RASSF1A* promoter hypermethylation reduces *RASSF1A* mRNA expression, which directly affects its function in the Ras-MAPK pathway, and is one of the important factors leading to LUAD progression (42).

## Conclusion

In conclusion, the methylation levels of CGIs in *SHOX2* and *RASSF1A* promoter regions are increased in early-stage disease, and may be useful as diagnosis biomarkers of early LUAD. *SHOX2* and *RASSF1A* promoter methylation was associated with abnormal folic acid metabolism and DNA instability, which may affect DNA replication and repair, apoptosis and tumor immunity. However, due to the limited number of patients in the NJDT cohort, the diagnostic potential of the combined *SHOX2* and *RASSF1A* promoter methylation assay in early LUAD is still incomplete. Another limitation of this study was that we only analyzed the CGIs in the promoter regions of two genes, and our study lacks the exploration of non-promoter and gene-body CGIs which can also affect gene expression. Therefore, the influence of abnormal methylation of these two genes on their mRNA expression needs to be further discussed, and the mechanisms of their participation in LUAD occurrence and development merits further evaluation. We hope that our research will facilitate the screening and diagnosis of early-stage LUAD patients and provide knowledge of tumorigenesis mechanisms and drug development.

## Data Availability Statement

The datasets presented in this study can be found in online repositories. The names of the repository/repositories and accession number(s) can be found below: https://db.cngb.org/search/project/CNP0002665/.

## Ethics Statement

The studies involving human participants were reviewed and approved by Ethics Committee of Nanjing Drum Tower Hospital. The patients/participants provided their written informed consent to participate in this study.

## Author Contributions

HG takes responsibility for all aspects of the reliability and freedom from bias of the data presented and their discussed interpretation, drafting the article. JY, LH, WW are responsible for performing the IHC experiments and evaluating the results. YL, YH, JD, and MG collected samples and data. QY take responsibility for full text evaluation and guidance. All authors contributed to the article and approved the submitted version.

## Funding

Our research was funded by Medical Science and Technology Development Foundation, Nanjing Municipality Health Bureau (YKK21105), Projects of Modern Hospital Management and Development Institute, Nanjing University (NDYG2021007, NDYG2021012), Open Projects of Jiangsu Biobank of Clinical Resources (SBK202006002, TC2021B012), Jiangsu Biobank of Clinical Resources (BM2015004) and Doctoral Research Start-up Foundation, First Affiliated Hospital of University of Science and Technology of China (RC2021131).

## Conflict of Interest

The authors declare that the research was conducted in the absence of any commercial or financial relationships that could be construed as a potential conflict of interest.

## Publisher’s Note

All claims expressed in this article are solely those of the authors and do not necessarily represent those of their affiliated organizations, or those of the publisher, the editors and the reviewers. Any product that may be evaluated in this article, or claim that may be made by its manufacturer, is not guaranteed or endorsed by the publisher.

## Supplementary Material

Supplementary Figure 1 The interaction of positive cases of the individual *SHOX2* and *RASSF1A* promoter methylation assays. T_*RASSF1A*: *RASSF1A* promoter methylation assay on tumor samples; T_ *SHOX2*: *SHOX2* promoter methylation assay on tumor samples; P _ *RASSF1A*: *RASSF1A* promoter methylation assay on the matched paracancerous samples; P_ *SHOX2*: *SHOX2* promoter methylation assay on the matched paracancerous samples.

Supplementary Figure 2 Presentation of clinicopathologic data and detection results of patients in combination*_*met (+) and combination*_*met (-) groups from the NJDT cohort. T_*RASSF1A*: *RASSF1A* promoter methylation assay on tumor samples; T_*SHOX2*: *SHOX2* promoter methylation assay on tumor samples; T_Combination: The combined promoter methylation assay of *SHOX2* and *RASSF1A* on tumor samples; T&P _*RASSF1A*: *RASSF1A* methylation assay on tumor and matched paracancerous samples; T&P_*SHOX2*: *SHOX2* methylation assay on tumor and matched paracancerous samples; T&P_Combination: The combined promoter methylation assay of *SHOX2* and *RASSF1A* on tumor and matched paracancerous samples; *SHOX2*_ct: the promoter methylation C_T_ values of *SHOX2* detected by qPCR; *RASSF1A*_ct: the promoter methylation C_T_ values of *RASSF1A* by qPCR.
