# Supplementary figures and images for: The Diagnostic Potential of SHOX2 and RASSF1A DNA Methylation in Early Lung Adenocarcinoma
Source: Front Oncol. 2022 Jun 28;12:849024. doi: 10.3389/fonc.2022.849024 (PMC9273978; doi:10.3389/fonc.2022.849024)

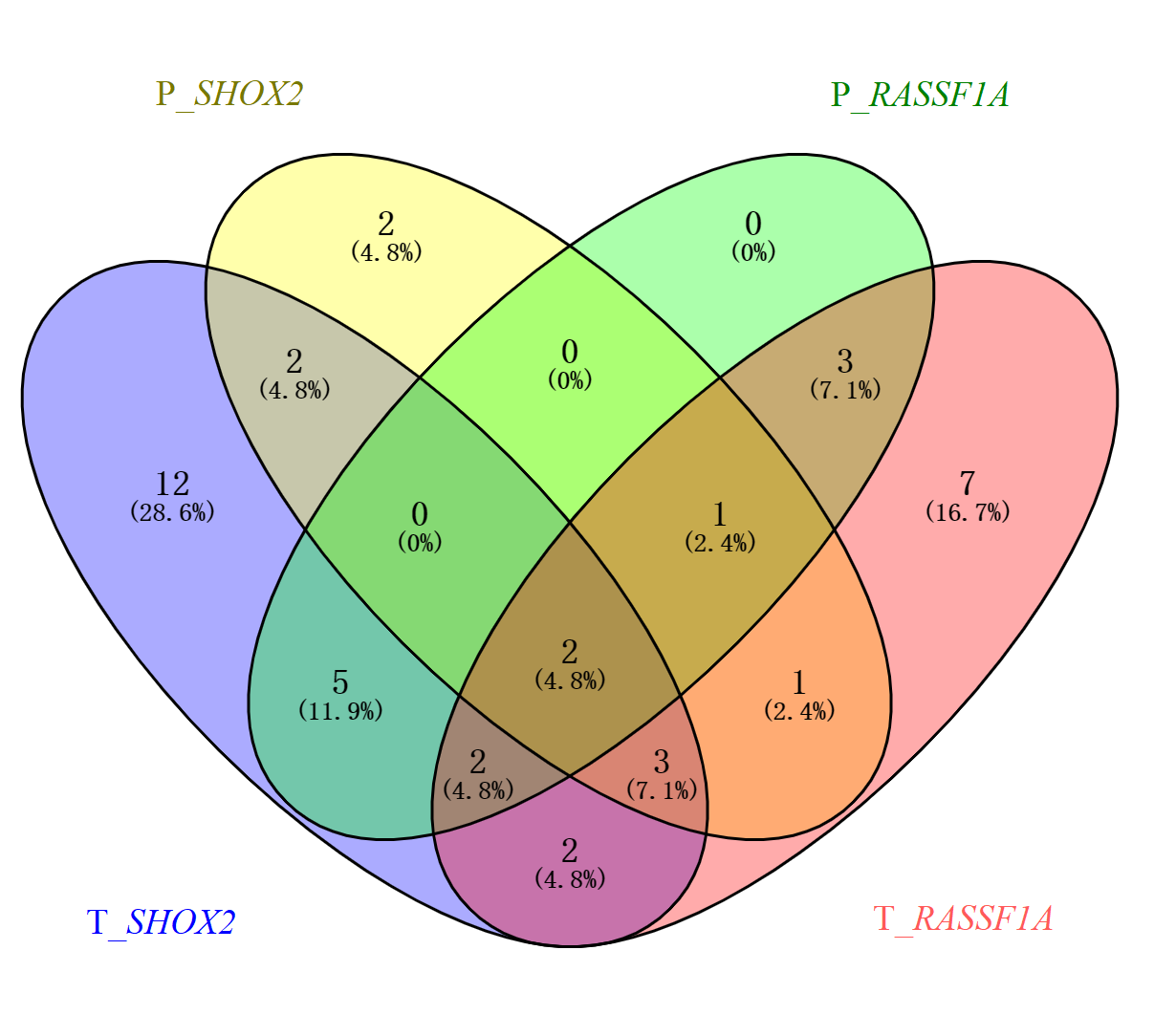

Supplement: Supplementary Figure 1 — The interaction of positive cases of the individual SHOX2 and RASSF1A promoter methylation assays. T_RASSF1A: RASSF1A promoter methylation assay on tumor samples; T_ SHOX2: SHOX2 promoter methylation assay on tumor samples; P _ RASSF1A: RASSF1A promoter methylation assay on the matched paracancerous samples; P_ SHOX2: SHOX2 promoter methylation assay on the matched paracancerous samples. [file Image_1.tif]

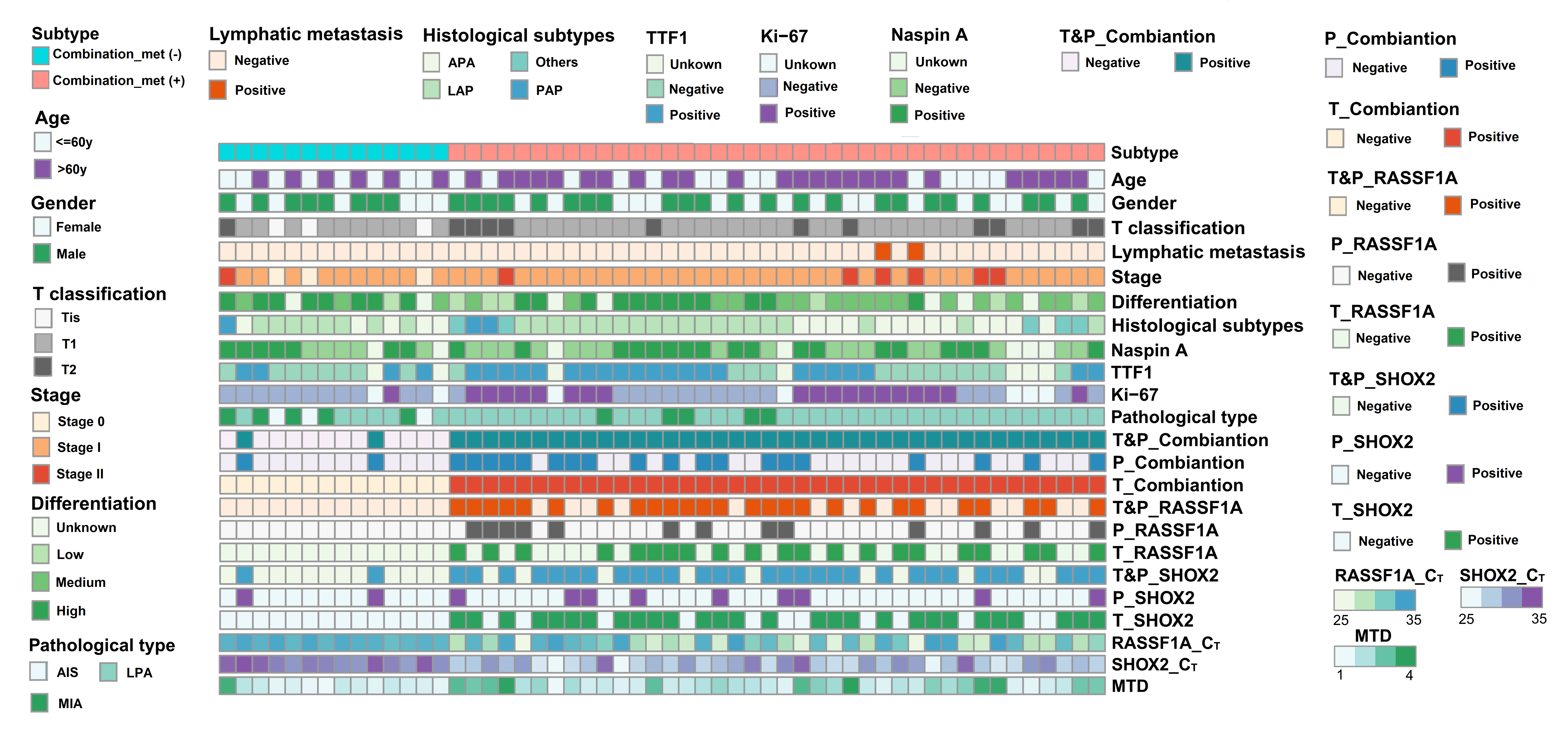

Supplement: Supplementary Figure 2 — Presentation of clinicopathologic data and detection results of patients in combination_met (+) and combination_met (-) groups from the NJDT cohort. T_RASSF1A: RASSF1A promoter methylation assay on tumor samples; T_SHOX2: SHOX2 promoter methylation assay on tumor samples; T_Combination: The combined promoter methylation assay of SHOX2 and RASSF1A on tumor samples; T&P _RASSF1A: RASSF1A methylation assay on tumor and matched paracancerous samples; T&P_SHOX2: SHOX2 methylation assay on tumor and matched paracancerous samples; T&P_Combination: The combined promoter methylation assay of SHOX2 and RASSF1A on tumor and matched paracancerous samples; SHOX2_ct: the promoter methylation CT values of SHOX2 detected by qPCR; RASSF1A_ct: the promoter methylation CT values of RASSF1A by qPCR. [file Image_2.tif]
